# Toward agenda 2030 in education: policies and practices for effective school leadership

**DOI:** 10.1007/s10671-023-09341-8

**Published:** 2023-04-08

**Authors:** Norma Ghamrawi

**Affiliations:** College of Education, Qatar University, Doha, Qatar

**Keywords:** Instructional leadership, Agenda 2030, Educational reform, School leadership, School improvement, Education quality

## Abstract

School leadership has been described as a key target for leveraging the quality of education in relation to sustainable development goal (SDG4) that seeks to ‘ensure inclusive and quality education for all and promote lifelong learning’ as per UNESCO 2030 agenda. This study provides a policy review of school leadership in the State of Qatar, as a case study, but carries out discussions within the global thrive for meeting the demands of Agenda 2030 to achieve quality for public education. It utilized a research instrument developed by UNESCO’s Division for Policies and Lifelong Learning Systems in Paris. The study used personal interview surveying, also called face-to-face surveying, and was completed with high-level policymakers at the Ministry of Education and Higher Education (MOEHE). It consisted of four sections that addressed: (1) the regulatory frameworks governing school leadership; (2) the professional development opportunities offered to school leaders; (3) the degree the school leadership profession was attractive; and (4) the procedures followed to appraise school leaders. Findings underscore the critical role played by school autonomy, instructional leadership, and governance in responding to Agenda 2030. The discussion contributes to the global discourse in meeting the requirements of Agenda 2030.

## Introduction

School leadership has been topping education policy schemata since the launch of UNESCO’s Agenda 2030. According to the UNESCO (2016), school leadership is a key priority for achieving its fourth Sustainable Development Goal, ‘ensuring inclusive and equitable quality education and promoting lifelong learning opportunities for all.’ This came after twenty-five years of the Education for All (EFA) movement, which emphasized student access, enrollment, and retention (Hopkins, 2015). The UNESCO’s shift from focusing on teachers, to focusing on school leaders was justified by the massive research evidence collected on the critical role played by school leaders in terms of school improvement (Schleicher, 2015).

Moreover, this shift was also inspired by the decision made earlier by the Organization for Economic Cooperation and Development (OECD) to invest in school leadership as means for school improvement for member states (Hopkins, 2015). The OECD has largely emphasized the importance of effective school leadership as a key for any educational reform (Guthrie et al., 2022; Jerrim & Sims, 2022; Pont et al., 2008). This is because school leadership has impact on the overall school climate (Gómez-Leal et al., 2022; Velarde et al., 2022; Watson, 2009), influences teachers’ motivation (Meyer et al., 2022; Tan et al., 2022; Thomson & Hillman, 2019), and improves the efficiency and equity in schools (Leithwood et al., 2020; Mayger & Provinzano, 2022).

As such, countries worldwide are working toward leveraging the quality of education by 2030 through focusing on school leadership. The targeted school leadership has been described in-depth according to the literature cited by both the OECD and the UNESCO. In brief, school leadership is effective when it is collaborative and inclusive (Council of the EU, 2017; Ketikidou & Saiti, 2022), recognizes the different talents and capacities of staff (Gonzales et al., 2022; Leithwood et al., 2020), distributes and shares power and authority (Ghamrawi, 2011; Ghamrawi & Tamim, 2022; Thien & Chan, 2022), and supports in the allocation teaching innovations for teachers (Hallinger, 2010; Tan, 2022).

In this line, the UNESCO’s Division for Policies and Lifelong Learning Systems in Paris has developed an instrument to help countries assess the state of affairs of school leadership to support their endeavors in responding to Agenda 2030. The knowledge of the status quo is the real first step toward setting plans for development. The researcher was involved in piloting it in two countries before endorsement.

This study examined the existing status quo pertaining school leadership in the State of Qatar, highlighting the degree the regulatory frameworks supported the notion of distributed and instructional leadership; the professional development offered to school leaders; the appraisal scheme for school leaders; and the degree school leadership was an attractive profession.

The overall purpose was to explore the degree the provision of school leadership in the State of Qatar and was in line with UNESCO’s Agenda 2030, which recommends school leaders:

- To serve the role of instructional leaders;
- To distribute leadership;
- To be continuously trained and offered professional development suited for their anticipated role; and
- To be attracted to the profession through monetary and non-monetary incentives (UNESCO, 2016)

## Importance of the study

Educational reform may not be attained in the absence of effective school leadership (Carrington et al., 2022; Day & Sammons, 2016). It is argued that an effective school leader is second to an effective classroom teacher in terms of influence on student learning outcomes (Leithwood et al., 2020). They create the organizational culture that is conducive for quality learning (Sun & Leithwood, 2015); upsurge the motivation of teachers and staff (Sortkaer et al., 2018); encourage risk-taking (Ghamrawi, 2013; Heffernan et al., 2022); and contribute to equity in schools (Mayger & Provinzano, 2022; OECD, 2016).

This study focused on exploring school leadership in the State of Qatar in the context of Agenda 2030, giving readers from Qatar, and other countries interested in Agenda 2030, an insight on the school leadership policies, that should be tapped on, in order to achieve quality education by 2030. The ramifications of findings from this study are discussed in the context of the global concern pertaining this agenda’s requirements.

Moreover, this study is also of interest to international readers, as it suffices the opportunity of exploring the application of UNESCO’s (Division for Policies and Lifelong Learning Systems in Paris) survey on one educational system. This is the first published work that utilizes this instrument. Readers may be interested in exploring the policy recommendations that were made to support effective response to Agenda 2030. In fact, Agenda 2030 is a global target; any researcher, policymaker or stakeholder involved in school improvement, and education reform would find value in it.

Thus, this study adds to the literature of school leadership in general and Agenda 2030 in particular. It should be an attractive read for policymakers interested in school leadership-based reform. It focuses on a combination of aspects of school leadership, not commonly focused on altogether, mainly its regulatory framework, professional development, appraisal and attractiveness as career. Finally, this study might be attractive to ministries of education who would like to assess their readiness for Agenda 2030 in what concerns school leadership.

## Research questions

This study was guided by the following research questions:

1. What regulatory frameworks govern school leadership in Qatar?
2. What professional development is offered to school leaders in Qatar?
3. To what degree is the school leadership profession attractive within the Qatari educational context?
4. How are school leaders in Qatar appraised?

## School leadership terminology

UNICEF (2020) estimates that there are at least a billion child, attending one form of a formal schooling system. This ‘mass’ formal education was not known before the last century (Gillard, 2018). Schools started receiving progressively demands expecting them to educate rounded children, who are well developed both academically and socially (Schechter et al., 2018). Recently, there have been additional demands on schools in light of the Covid 19 pandemic, which showed weak school resilience (Bellei et al., 2022), as they were inapt at handling the challenges of the crisis (Cairney & Kippin, 2022).

Central to this comes those who run schools, holding different names, and being referred to under varied titles such as school managers, head teachers, school administrators, and school leaders (Townsend, 2019). A study carried out by Gumus et al., (2018) covering all published work on school leadership between 1980 and 2014 showed an increasing interest in leadership models in educational research over time. Gumus et al. (2018) enlisted 14 educational leadership models in their review, including: distributed leadership, instructional leadership, teacher leadership, transformational leadership, curriculum leadership, technology leadership, transactional leadership, ethical/moral leadership, charismatic leadership, administrative/managerial leadership, strategic leadership, authentic leadership, visionary leadership, and servant leadership.

The four educational leadership models topping Gumus et al. (2018) list were, in order: (1) distributed leadership, (2) instructional leadership, (3) teacher leadership, and (4) transformational leadership. Gumus et al. (2018) review also showed that published studies focused on the above terminologies in relation to two broad aspects: organizational behaviors and student achievement. Interestingly, UNESCO’s Agenda 2030 is centered on both distributed and instructional leadership, considering them as primers for school reform (UNESCO, 2016).

## Prominent leadership models in educational research

One of the most researched leadership models in educational research is transformational leadership. It was transformed from the business sector to education by Ken Leithwood, in Canada, in 1980. With transformational leadership, the focus is on influencing subordinates by securing the vision and inspiration that are conducive for transcending their self-interests for the interests of the groups they belonged to (Berkovich, 2016; Gunter, 2016). The benefits of transformational leadership, as reported in the literature are numerous, focusing on the potential of this leadership model in stimulating and inspiring followers to achieve outstanding outcomes, while building their own capacities as leaders (Bass & Riggio, 2006; Hendricks & Scheerens, 2013).

However, transformational leadership is criticized for being too idealistic for practice, as it is built on the notion of a heroic leader, with extraordinary performance (Gronn, 2003; Gunter, 2016; Stevens, 2022). Moreover, some literature, such as Day and Sammons (2016), suggest that transformational leaders are inherently instructional leaders who get involved in learning and teaching, thus shaping and influencing leaners’ outcomes.

This leads to another highly addressed leadership model in the literature, instructional leadership. The instructional leader seeks to ‘influence conditions that directly impact the quality of curriculum and instruction delivered to students in classrooms’ (Hallinger, 2003, p. 338). Instructional leadership model is essentially focused on the establishment of clear vision, management of the instructional program, and the promotion of a positive school climate (Hallinger, 2015). In this line, instructional leadership is best viewed as an approach for safeguarding instructional time and maximizing its quality through providing incentives for learning and teaching (Neumerski, 2013), besides promoting high quality teacher professional development (Liu et al., 2021). This is in addition to maintaining high visibility in and between classrooms (Ma & Marion, 2021) and establishing and enforcing rigorous academic standards (Hallinger, 2015).

It should be noted that, distributed leadership is the mostly addressed leadership model in the literature. According to Spillane (2005), it is used interchangeably with ‘shared leadership,’ ‘collaborative leadership,’ ‘delegated leadership,’ and ‘dispersed leadership.’ In all cases, this leadership model is not restricted in one figure in the school, but rather dispersed to include staff at all levels (Harris, 2013; Thien & Chan, 2022). It has to do with power sharing (Hatcher, 2005; Özdemir et al., 2022), empowering staff (Jambo & Hongde, 2020), and building the capacity for improvement (Carrington et al., 2022; Harris, 2013).

## School leadership and school improvement

A massive number of published work considers school leadership as a pre-requisite for educational reform (Bush & Middlewood, 2013; Harris, 2013; Mette & Scribner, 2014; Harris & Jones, 2015; Day et al., 2016; Brooks & Normore, 2017; Castillo & Hallinger, 2018; Lochmiller, 2018; Myran & Sutherland, 2019; Leithwood et al., 2020; Anderson, 2021; Evans, 2022).

Distributed leadership has been recognized as means for organizational improvement (Hallinger & Heck, 2009) and increased student outcomes (Leithwood & Mascall, 2008). Leithwood et al. (2009) went into attributing the difference between low and high performing schools to the degree school leaders in those institutions managed to distribute leadership efficiently.

How school leadership fosters school improvement is best described by the functions outlined by Day et al. (2016). Day et al. (2016) suggest that effective school leaders often (1) shape a vision of success for all school members; (2) create growth-promoting collaborative cultures; (3) cultivate leadership in others; and (4) serve as instructional leaders. These findings corroborate with the vast literature on school leadership and school improvement.

In fact, the literature suggests that effective school leaders realize school improvement by leading on teaching and learning, setting expectations about the school’s learning practices and a focus on improving student success (Leithwood, 2021). Moreover, they tend to show a high level of continued professional development for the self, and for each member of the school, by creating cultures of empowered, self-directed learning, and responsibility (Doherty, 2021; Leithwood, 2021). In addition, effective school leaders secure school improvement by staying open to innovation and change (Sudibjo & Prameswari, 2021) and by advocating a drive toward new initiatives in the school environment (Acton, 2021). They also tend to invest in cultures of data-driven decision-making at all school levels, to ensure efficient operations for best learning outcomes for students (Leithwood, 2021). Finally, they liaise continuously with the wider school community (Mayger & Provinzano, 2022).

## School leadership in Qatar

Qatar embarked on an educational reform journey called “Education for a New Era’ in 2004, as a response to the education sector analysis carried out by RAND corporation which considered schools to lack the vision and mission, to be controlled by top-down hierarchal organizational structures, and to be dominated by teacher-centered classrooms (Nasser, 2017). As such the Qatari government developed the so called ‘independent schools’ (charter schools) aiming for increasing school autonomy and hence decentralization (Nasser, 2017).

However, this journey was concluded in 2016, under a massive barrage of stakeholders’ resentment as being imported from the west, with little sensitivity pertaining to the Qatari culture and identity (Romanowski & Du, 2020). According to Nolan (2012), “the extent of the challenge and the lack of clear guidelines undermined community confidence in the reform efforts” (p. 26). Thus, public schools in Qatar were again re-centralized (Abou-El-Kheir, 2017).

Within this context, the school leadership role was guided by Qatar National Professional Standards for Teachers and School Leaders (QNPSTSL), developed by the Education Queensland International (EQI) of Australia, which explicated the knowledge, skills, and dispositions required from teachers and administrators (Romanowski & Du, 2020). School leaders’ standards were originally seven, but were then downsized into 5, including: (1) Leadership and management the school strategically, (2) leading and managing teaching and learning within the school community, (3) achieving high standards for continuous improvement, (4) managing, leading, and developing individuals and teams, and (5) managing and leading school relationships with parents and the community.

## Qatar national vision 2030

According to the MOEHE website,^1^ ‘Qatar National Vision 2030 (QNV2030) aims to build the capacities of Qatari citizens by establishing world-class education system that offers exceptional opportunities for quality education and training’ (para. 1). In fact, Qatar has set for itself the year 2030 for joining world-class educational systems that rooted in creativity and innovation (Alshahrani & Ally, 2016).

This gives rise to an intersection between Qatar’s National Vision 2030 and UNESCO’s Global Agenda 2030. In fact, for Qatar to reserve a location for itself among leading countries in education, it needs to respond to UNESCO’s 2030 Agenda, which has been endorsed globally by world-class educational systems. In other words, to meet the requirements UNESCO’s Agenda 2030 and fulfill Qatar’s Vision 2030, policymakers should be working toward endorsing distributed and instructional leadership models within Qatar’s public school system, offering high quality professional development for school leaders, ensuring that the school leadership position is quite attractive among other careers, and securing an effective appraisal system. This is the core of what this study explored.

## Research methodology

### Research design

This study was qualitative in nature aiming at gaining deep empathetic understanding of the status quo of school leadership in Qatar. It utilized a personal interview survey, also called face-to-face survey (Schröder, 2016) in order to explore the responses of the people to gather more and deeper information (Zhang et al., 2017). This method is often utilized when a specific target population is involved, and the concern is the collect data from its source to build valid findings based on it (Fowler, 2013; Zhang et al., 2017).

### The sample

Participants in this study were three high-level policymakers at the MOEHE, constituting the population of high-stake decision-makers at the MOEHE. All other staff at the Ministry execute orders and decisions made by these three key people. They were known for their involvement in the policy development and application of those policies in schools. When approached, those candidates welcomed taking part in the study without any hesitation.

### The research instrument

This study utilized UNESCO’s (Division for Policies and Lifelong Learning Systems in Paris) survey. The researcher piloted this instrument earlier in two other countries in the region. However, this is the first time; this instrument is being used formally in a published work. The instrument consists of four sections that address: (1) the regulatory frameworks governing school leadership in Qatar; (2) the professional development opportunities offered to school leaders; (3) the degree the school leadership profession is attractive within the Qatari educational context; and (4) the procedures followed to appraise school leaders. It should be noted that for each claim made during the interviews, and the interviewees were asked to provide a proof to support their claims. That is why the instrument was shared with them prior to the actual interview, so that they could be well ready for sharing such proofs and evidences. Finally, the answers collected from all three interviewees were consolidated in the tables presented in this study. The completed tables were shared with them upon completion, and no objection was received back. In all cases, there were little to no difference in ratings received from the three participants, as they worked as a team across all initiatives. When there was a discrepancy, which mostly were a point difference in the scale, the researcher reported an average which shifts the rating toward the selection made by two of the participants as opposed to the third.

### Gaining access

Initially, the researcher approached the head of the Division for Policies and Lifelong Learning Systems in UNESCO headquarters in Paris, to gain the permission to use the instrument formally in a study. She received positive confirmation, and encouragement to use it. After that, she approached the MOEHE to check their interest in this study.

The MOEHE received an application that included a copy of the research instrument, that clarified the purpose of the study, how data was going to be stored and used, and giving assurances of anonymity of participants. Upon consent, the researcher gained the approval of the ethics committee at Qatar University.

The personal interviews were all carried via Microsoft Teams, and the MOEHE requested to have a copy of the full data collected. As such, the researcher emailed the MOEHE with a complete copy of the instrument alongside the responses collected per each item. No objection was received back for the MOEHE pertaining data.

### Data analysis

Data collected from the personal interview survey was analyzed using content analysis, which is widely used for interpreting meaning from text data, and thus deriving important aspects of the content (Stemler, 2015). It was used for ‘making systematic, credible, or valid and replicable inferences from text’ (Drisko & Maschi, 2016). ‘Deductive content analysis is beneficial for testing concepts, categories, theories or any conceptual structure in a new context’ (Kyngäs & Kaakinen, 2020, p. 9). In other words, deductive content analysis shares similar features of inductive content analysis used in qualitative research, with a key difference that deductive content analysis is applied with a priori theoretical knowledge as the starting point. The research questions and data collection are both influenced structures around such prior knowledge. In addition, another key difference between inductive and deductive content analysis is that deductive content analysis is guided by structured analysis matrix (Kyngäs & Kaakinen, 2020; McKibben et al., 2020). As in inductive content analysis, the reporting of results should be structured according to the identified concepts, categories and/or themes (Kyngäs & Kaakinen, 2020, p. 9). In this study, the researcher utilized deductive analysis of data based on four pre-determined categories governing school leadership: regulatory frameworks, professional development, attractiveness of the position, and appraisal procedures.

## Findings and discussion

### School leadership model

The level of school governance that was responsible for the decision-making in key aspects of schooling was the first addressed issue. Findings from the personal interview survey offered the data presented in Table 1.

**Table 1:** Decision-making classified per governance level

Items	MOEHE	Schools
Recruiting/appointing and promoting principals	X	
Dismissing or suspending principals from employment	X	
Establishing principals’ starting salaries including setting pay scales	X	
Recruiting appointing and promoting teachers	X	
Dismissing or suspending teachers from employment	X	
Establishing teachers starting salaries including setting pay scales	X	
Deciding on budget allocation and use of resources within school	X	
Defining curricula, including course content	X	
Determining teaching methods	X	X
Determining school organization and schedule	X	X
Determining student assessment policy	X	
Deciding on textbooks and learning materials	X	

As Table 1 shows, the key decision-making is made at the level of the MOEHE. The MOEHE monitors the details of all the key decisions taken by schools. There is a very small margin for decision-making left out for school leadership, covering only the teaching methods, and the development of schedules. However, even in what relates to those decisions, it is not left out completely for schools, as the MOEHE might suggest things, and requests to see those schedules.

‘We are keen that schools work under the same ethos and toward the same vision, so by making decisions pertaining to teacher recruitment, dismissal, budgeting; we ensure homogeneity of practices. School principals and their vices can make some decisions pertaining to teaching protocols’ (P2).

As such, school leadership is far from being distributed, delegated, or shared by the MOEHE to school leaders. All key decisions within the school system are dictated to school leaders, rather than giving them the margin of freedom to practice autonomy. Research suggests that school leadership that lacks autonomy is more likely to avoid distributing or sharing leadership (OECD, 2016). PricewaterhouseCoopers (2007) explained that distributed leadership in schools could never prevail when there are legal or regulatory barriers that inhibit them, such as the lack of autonomy.

The literature cited by the UNESCO in presenting Agenda 2030 highly emphasizes the importance of distributed forms of school leadership and autonomy (Mac Ruairc, 2010). Moreover, the OECD, which was the primer in inspiring UNESCO’s Agenda 2030, has linked higher degrees of autonomy and distributed forms of school leadership, to higher student outcomes on PISA (Mac Ruairc, 2010).

A further investigation into the degree school leaders endorsed instructional leadership model may be inferred from the key tasks that were carried by school leaders, as presented in Table 2.

**Table 2:** Key tasks carried out by school leaders

Key tasks	Percentage* (rough estimate)	Very High	High	Moderate	Nil
Administrative and leadership tasks and meetings *Including human resource/personnel issues, regulations, reports, school budget, preparing timetables and class composition, strategic planning, leadership and management activities, responding to requests from district, regional, state or national education officials*	60	☐	☐	☒	☐
Curriculum, and teaching-related tasks and meetings *Including developing curriculum, teaching, classroom observations, student evaluation, teachers mentoring, and professional development*	10	☐	☐	☒	☐
Student interactions *Including counseling and conversations outside structures learning activities and discipline*	10	☐	☐	☒	☐
Interactions with local and regional community, business, and industry	10	☐	☒	☐	☐
Parent or guardian interactions *Including formal and informal interactions*	10	☐	☒	☐	☐

*These percentages are rounded figures of the rough estimates provided by all participants

Table 2 shows that the majority of the time of school leaders was devoted to administrative and leadership type of tasks and meetings. Time allotted for curriculum and teaching-related tasks and meetings, student interactions, and interactions with parents and the local community received less than half of the school leaders’ time.

‘We believe that we have appointed vice principals and subject coordinators to carryout tasks related to teaching and student learning. School principals have massive amount of administrative work to carry out, and are very busy showing up in classrooms and attend classes’ (P1).

Focusing on the time allocated for involvement in the learning and teaching at school, the figure is incredibly low, compared to figures recommended for instructional leaders, which suggest 50% or more of his/her time (Fink & Resnick, 2001; Hayes & Irby, 2020). This leads to the conclusion that there are few chances for Qatari school leaders to practice instructional leadership, as they are soaked in administrative type of tasks.

### Selection criteria for the school leader position

The eligibility criteria for the selection of the school leader for the position are presented in Table 3.

**Table 3:** Selection criteria for the school leader position

Eligibility criteria	Yes	No
Academic qualification (minimal bachelor degree)	X	
Registration as a teacher by a regulatory authority		X
Evidence of good teaching experience		X
Experience of in-school wide leadership and management responsibilities		X
Experience as education advisor or counselor or mentor		X
Teaching experience in basic education		X
Selection follows a competitive process	X	
Selection process of school leaders is based on national qualifications standards	X	
Selection process is gender-based	X	
Selection process is limited to Qatari nationals	X	
Others: 7 years within the school system		

According to Table 2, two factors are essential for eligibility for the school leadership position: the academic degree and the completion of 7 years within the school system. This could or could not include any past leadership or managerial experience, or a classroom teaching position. In other words, there are no specifications pertaining the nature of experience completed within those years.

‘We do not require school principals to bear a prior teaching experience. While many could have served as teachers before taking over the school leadership role, decrees do not consider a condition for becoming a school principal’ (P3).

This finding is not aligned with Agenda 2030, which puts great emphasis on the ability of the school leader to serve as an instructional leader, by being a teacher before occupying the role of the school principal (Michelsen & Wells, 2017). In fact, according to UNESCO (2016), distributed leadership works best when coupled with pedagogical or instructional leadership, which focuses on student learning, and is best achieved when the school leader bears a professional teaching experience in his/her career path prior to occupying the formal leadership position as a school leader (UNESCO, 2016).

On the other hand, selection of the school leader follows a competitive process in Qatar, whereby those interested in the position; follow a preliminary program to assess basic knowledge and skills required for the position. Candidates are given scores, based on which, the search committee promotes them for an interview. The interview is centered on the Qatari National Professional Standards for School Leaders (QNPSSL), which will addressed in the next section in this paper.

‘All candidates enroll in a leadership program and get scored based on our national standards which gives them eligible for the selection interview. Next these standards will shape the lives of principals as they are assessed based on them each year, if selected for the job. Another factor we take into consideration is a quota for male/female school principals as we match principals’ genders with student genders in secondary’ (P2).

This goes in line with the international literature underling Agenda 2030, which suggests that school effectiveness is enhanced when the national qualifications standards constitute the basis for the selection of school principals (Pont et al., 2008). In the same vein, the QNPSSL are used to dictate job descriptions for school leaders (Pont et al., 2008) and are used as assessment criteria in licensing procedures (Smylie & Murphy, 2018).

Furthermore, government schools in Qatar are gender-segregated, meaning that there are boys-only schools and girls-only schools. As such, except for primary schools, male school leaders lead the boys-only schools, while female school leaders lead girls-only schools. Consequently, the selection is gender-based. While promoting gender equality is one of the internationally agreed goals and targets of the Agenda 2030, this finding does not contradict this direction because it ensures good space for women in leadership positions. Given the fact that almost 50% of government schools in Qatar are girls-only schools (QNB, 2022), then, this gives a highly recommended figure in terms of women recruitment for leadership positions. Women in leadership positions have been noted to do better at persuasion (Von Hippel, 2011) and establishing effective inclusive teams (Slaughter, 2012).

Finally, a note to mentions is that Qatari nationals are given priority during the selection process over non-nationals, as part of the ‘*Qatarization initiative,*’ a basic pillar of Qatar Vision 2030, which was devised by the government in order to increase the number of Qataris employed in both public and private sectors. This relates to the fact that Qataris constitute only 10% of the overall population of the country,^2^ as it is home for a huge number of expatriates.

### Qatar’s national professional standards for school leaders (QNPSSL)

As stated earlier, Qatar has a set of national professional standards for school leaders (QNPSSL) that prescribe the effective functioning of school leaders covering 5 domains, already presented in the review of literature section. These standards are focused on maintaining school direction, establishing organizational development, giving life to data, and supporting student-oriented classrooms.

The mapping of the QNPSSL against international standards, presented in Table 4, shows good alignment with international practice. This was an expected finding, as a major critique for the QNPSSL was that it was imported from the west (Ellili-Cherif et al., 2012). However, participants in the study were asked to illustrate how standards were being practiced, to make a solid judgment that does not rely only on published documents, but rather on how such standards were meant to be practiced from the highest policy authority in the country.

**Table 4:** Mapping QNPSSL against core professional leadership and management practices acknowledged by agenda 2030

Key areas	Core professional leadership and management practices	Very strong	Strong	Moderate	Nil
Shaping the future	Creating a shared vision and strategic plan for the school (in collaboration with governing body) that motivates staff and others in the community			X	
Leading learning and teaching	Raising the quality of teaching and learning for students’ achievement. This implies setting high expectations, monitoring, and evaluating the effectiveness of learning outcomes		X		
Developing self and working with others	Building effective relationships and building a professional learning community through performance management and effective professional development for staff		X		
Managing the organization	Improving organizational structures through self-evaluation, organization, and management of people and resources in order to build capacity across the workforce and deploy cost effective resources	X			
Security accountability	Principals are accountable to pupils, parents, caregivers, governors, the local authority and the whole community to provide a high-quality education for promoting collective responsibility within the whole school community and for contributing to the education service more widely			X	
Strengthening community	Creating links and collaborating with other schools, parents, caregivers, and other agencies to share expertise and ensure children’s well-being	X			

Table 4 shows high quality national professional standards for school leadership; however, as stated earlier, they have received massive critiques as being western imports that were not rooted in the Qatari context and culture (Ellili-Cherif et al., 2012; Nasser, 2017; Romanowski & Du, 2020). Empirical findings have shown that school leaders believed that the QNPSSL included ‘ambiguous terminology and procedures, ignored local educators’ input, and provided unrealistic expectations of society, lacked consistency, and created resistance on the part of educators’ (Ellili-Cherif et al., 2012, p. 471).

### Professional development for school leaders

School leaders in Qatar seem to enjoy a wide spectrum of professional development activities sourced by the MOEHE. Table 5 summarizes how school leaders are prepared and supported to ensure effectiveness in relation to professional standards.

**Table 5:** School Leaders’ Preparation for the Position

Items	Yes	No
There are institutions offering pre-service training for school principals	☒	☐
There are institutions offering in-service training for school principals	☒	☐
Induction programs are systematically provided for new school principals	☒	☐
School Principals follow a governmental licensing program	☒	☐
New school principals are systematically mentored by practicing principals	☐	☒

School leaders are offered capacity building prior to taking over the role, and across their career, in order to grow professionally along their years of service. Training is offered by the MOEHE’s Training and Development Center, or is outsourced through it. In all cases and stages, great support and incentives are offered to school leaders for them to attend training. Incentives include financial support and leave allowances from school. This is a high recommendation noted through the research cited for Agenda 2030 by the UNESCO (2016). However, the system was missing on coaching and mentoring opportunities that would bring rigor and sustainability as per OECD (Berkovich & Benoliel, 2021).

‘We believe school principals do not need to be coached or mentored by other school principals because we do offer them all the professional development they need to succeed in their roles, from the source, and not cascaded through other principals’ (P3).

Moreover, the investigation of the key barriers noted by the UNESCO (2016) confronting the professional development of school leaders (Table 6) shows that except for the teaching experience, the barriers are nil. In fact, professional development is offered free of charge and is safeguarded by financial incentives. In addition, school leaders are given time-off their schools to attend those professional development opportunities when they arise.

‘Our school principals pay nothing to be trained and grow professionally. Contrarily, they are allowed to take a paid-off from work to attend workshops’ (P1).

**Table 6:** Barriers confronting School Leaders Professional Development

Items	Very high	High	Moderate	Nil
Many principals do not have the pre-requisites (e.g., qualifications, teaching experience, seniority)	☐	☐	☒	☐
Professional development is too expensive/unaffordable	☐	☐	☐	☒
Government does not provide budget for professional development	☐	☐	☐	☒
Professional development conflicts with principals’ work schedule	☐	☐	☐	☒
There is no relevant professional development offered to principals	☐	☐	☐	☒
There are no incentives for participating in such activities	☐	☐	☐	☒

## School governance

Government schools do not have official school management teams, but do have school governing boards (SGBs). The description of the composition of the SGB is presented in Table 7.

**Table 7:** School governing bodies in government schools

Items	Yes	No
Representatives of national authority	☐	☒
Representatives of municipality/regional authority	☐	☒
Representatives of local authority	☐	☒
School principals	☒	☐
School administrative personnel	☒	☐
Teachers	☒	☐
Students	☐	☒
Parents or guardians	☐	☒
Trade Unions	☐	☒
Representatives of business (labor market institutions) or private sector	☐	☒
Representatives of faith-based organizations	☐	☒
Representatives of civil society organizations, communities, NGOs, etc.	☐	☒

As Ranson et al. (2005) proposed, there are distinctive types of governing bodies based on their scope of influence; the balance of power; the margin for decision-making. (1) *Governance as a deliberative forum,* which are more of a gathering for members taking the shape of a club led by the school leader; (2) *Governance as a consultative sounding board,* where members approve policies brought the school leaders; (3) *Governance as an executive board,* where the board participates in the budgeting, staffing, and the infrastructure of building; and (4) *Governance as a governing body*, where the board provides strategic leadership for the school.

The SGB for the Qatari government schools seems to be at the lower spectrum of level one of Ranson et al. (2005) categorization.

‘Schools enjoy a school governing body that brings together the principal, his/her team, subject coordinators. They ensure the effective functioning of the school’ (P2).

In fact, it may be argued that the name has mistakenly be given to the board as a governing board, as it is more likely to be a management team as it is purely internal to the school. According to Agenda 2030, the recommendation is to have the SGB’s to fall into the fourth category of Ranson et al. (2005). However, this may not be achieved unless the recommendation to leverage school autonomy is in place.

### Attractiveness of the school leader position

The school leadership position in Qatari government schools is a highly attractive position as presented in Table 8.

**Table 8:** Attractiveness of the school leader position

Items	Yes	No
A major proportion of teachers (beyond 50%) aspire to school leadership position?	☒	☐
A principal’s average basic salary is significantly greater than a teacher’s average basic salary	☐	☒
Additional financial incentives are offered to school leaders in different areas	☒	☐
Other incentives (e.g., Prizes, rewards, recognition) exist to encourage best performer school principals	☐	☒
School principal salary is lower than that of other mangers in the public sector with similar qualifications and experience	☐	☒
School principal salary is lower than that of other mangers in the private sector with similar qualifications and experience	☐	☒
School principal salary is the same as that of other mangers in the public sector with similar qualifications and experience	☒	☐
School principal salary is the same as that of other mangers in the private sector with similar qualifications and experience	☒	☐
School principals have a respectable and prestigious social status in the society	☒	☐

As Table 8 shows, applicants for the position are quite high because the position is respectable and bears a prestigious status within the Qatari society. While the rank and salary scale stipulate the basic salary for the school leaders, they are entitled 35% allowance of the basic salary. Moreover, they are equally paid as other managers in both the private and public sectors of similar requirements. Exceptions include industry and petrol domains.

‘Becoming a public school principal in Qatar is a dream to many, because it brings along both social status and excellent pay. I can prove to you by figures that the pay is number one in the world’ (P3).

Finally, there are no prizes to distinguish high performing school leaders, yet there are prizes given to schools based on contests in specific areas. This leads to the conclusion that school leadership is an attractive position as per recommendations of the Agenda 2030 for Education.

### School leaders appraisal

All school leaders in government schools need to be appraised as per national regulations.

‘School principals are appraised by ministry assigned experts, and it is secrecy, where information is not disclosed except for the principal’ (P1).

So, appraisal is a task solely undertaken by the MOEHE, and more details are presented in Table 9.

**Table 9:** School leaders’ appraisal

Items	Yes	No
Only the Ministry of Education is responsible for appraising school principals	☒	☐
There is a national or state requirement to appraise school principals	☒	☐
School leaders’ appraisal is based on national professional standards	☒	☐
There are established policy frameworks that set out procedures for appraisal of principals	☒	☐
School principals are appraised on a mandatory periodic basis according to procedures defined through a central/state framework	☒	☐
Appraisal is required as an employment-related regulation in the case of re-appointing school principals into their position	☒	☐
There is an institutionalized mechanism for appraisal and feedback of school principals by their counterparts (peer review), in addition to appraisal and feedback by education inspectors	☐	☒
There is an institutionalized mechanism to collect and analyze the information from appraisal and feedback processes in order to spread effective leadership practices among school principals	☐	☒

Table 9 suggests that the regulatory framework for school leaders requires mandatory appraisal for school principals. As recommended by UNESCO (2016), this appraisal is built around the QNPSSL and is established against established policy frameworks. The details of the frequency of appraisals and their procedures are public and are available on the MOEHE website. However, contrary to Agenda 2030 recommendations, the appraisal is top-down and is limited to the sphere of authority of the MOEHE. Moreover, no sharing of data, findings, or success stories between members of the school leaders’ community is in place.

## Summary

This study presented a panoramic picture for school leadership in Qatari governmental schools. It showed that schools principals were more of managers than being leaders. Their time was absorbed with a relatively large volume of administrative tasks that left them with little time for practicing instructional leadership, which has been considered a core for leveraging education quality as per Agenda 2030.

Moreover, school autonomy was very limited. In fact, almost all essential leadership roles that characterize school leaders according to the international literature were taken over by the MOEHE. This left a very narrow margin for them to practice leadership. Consequently, it can be inferred and argued that distributed leadership was unlikely to prevail in those schools.

In addition, the so-called school governing bodies did not offer real governance for the school. The constitution of the SGB’s is far from international practice and resembles more internal leadership teams for schools. This left the school leader accountable only in front of MOEHE. Besides, school leaders were appraised based on a clearly defined framework. However, that was unidirectional, involving the MOEHE only.

On a brighter side, the role of school leaders in Qatar was governed by national professional qualifications (QNPSSL) which were used to specify job descriptions, select school leaders and appraise them. Despite receiving criticism through empirical studies for being imported from western educational contexts, the systematic use of those standards is commended.

In the same line, school leaders in Qatar seemed to be well catered for in terms of professional development and capacity building. In fact, the MOEHE sets continuous professional growth opportunities for school leaders, which they attend during work hours. However, the element of collegiality and learning communities remains very limited. In fact, the system misses on investing in collaborative models for professional development that encourage school leaders to share expertise and develop as learning communities.

Finally, the school leadership position was quite attractive, receiving social respect, and securing appreciable allowances. The selection criteria for school leaders were quite competitive, supported women in leadership positions, yet missed on the importance of teaching experience which remained non-mandatory.

## Conclusion

As stated earlier, the purpose of this study was to review policies underpinning school leadership in governmental schools in the state of Qatar, in the context of Agenda 2030. However, this endeavor is a global one, and countries interested in leveraged education quality are racing with time to achieve that in full or in parts, by the set date.

Agenda 2030 in education suggests that schools need to be autonomous, embrace distributed models of leadership, endorse instructional leadership, strengthened by school leadership teams, and safeguarded by functional school governing bodies. Moreover, the school leadership position should be an attractive position, respected in the society, giving women chances to lead, and is preceded by sufficient years of teaching experience in classrooms. In addition, school leaders should be selected based on a set of national professional standards adopted by the country reflecting best international practice. These should constitute the corner stone for continuous professional development (CPD) that caters for actual leaders’ needs. School leaders need to receive support and incentives to get involved in CPD opportunities. Nevertheless, school leaders should be appraised based on the national professional standards, regularly and according to clear plans.

The Qatari case study has shown huge efforts set forth by the MOEHE; however, despite that, they were still far from Agenda 2030 goals. All downsides can be summarized under one title: *lack of school autonomy*. Because schools lacked autonomy, the role of the school principal was limited to management and hence, fulfillment of agendas, plans, and activities set forth for schools by the MOEHE. This had the tendency to narrow down school leaders’ time available for catching up with instruction and hence, inhibiting their roles as instructional leaders.

The lack of school autonomy also explains the lack of functional and rigorous overarching school governing bodies. Effective school governing bodies often render the school leader accountable to them, besides being accountable to ministries of education. With non-autonomous schools, school leaders report only to the central educational authority, which is in this case the ministry. This makes schools miss on a highly progressive element for school improvement, which is student and parents’ voices.

In the same vein, the lack of school autonomy influences the type of professional development offered to schools. In this case, it tends to get decided by the central educational authority and to be unidirectional. With an authoritarian, top-down approaches to school management by such authorities, school leaders act out as receivers of training, rather taking part in shaping it. This also has the effect of inhibiting collaborative and collegial forms of cooperation between school principals. The same argument applies for the case of appraisal, which is also unidirectional.

A similar justification can be made for the criticism noted in the literature on the QNPSSL as being imported and non-contextual. When schools are expected to be managed rather than led, school leaders and their teams are less likely to be invited to share in decisions with the central educational authority. This renders standards non-contextual and even ambiguous as described in the literature.

Findings from the Qatari context should not be read apart from its history in education reform. In fact, as stated earlier, Qatar embarked on an educational reform “Education for a New Era’ in 2004 and developed ‘independent schools’ (charter schools) aiming for increasing school autonomy and hence, decentralization. However, because the reform was top-down, rather than being bottom-up, it was based on transferring elements from several western-based educational systems. The reform initiative did not achieve its goals, and the journey was concluded in 2016. It seems that this experience left policymakers in Qatar less confident in decentralization and in autonomous schools. Thus, strengthening authoritarian top-down management of schools by the MOEHE.

That is to say, countries interested in achieving education quality based on the recommendations of the research-based Agenda 2030 and should be focused on school autonomy and decentralization, in the first place. Static education environments are not conducive for school improvement. Schools need to enjoy a margin of autonomy in order to be able to respond to challenges confronting them, and to societal demands. However, careful attention should be made, as school autonomy is not a guarantee for school improvement. It should come with a package that includes clear role expectations centered on student learning, training on the role, and with an accountability scheme to safeguard it.

One compromise for countries that are hesitant to adopt school autonomy, as is the case of Qatar, could situate themselves in the middle. They can increase school autonomy by freeing their decision-making schemes, while centralizing both the governing standards and accountability requirements.

## Limitations, implications, and further research

It may be argued this study does not contribute to the literature of school leadership, because it utilized the case study methodology, using a small sample. However, the literature of case studies by prominent researchers such as Robert Yin, Sharan Merriam, and Robert Stake suggest that the case study methodology may be used to generate theories (Yazan, 2015). According to Siggelkow (2007, cited in Yazan, 2015), a single case study may be sufficient to contest a widely held view. The methods, instrument and data analysis approaches used in this study may be replicated in other contexts. Moreover, this study interviewed the population of policymakers (top three) actually involved in the immediate decision-making and policy making pertaining school leadership. As such, this study presents policymakers with a road map to evaluate their readiness for Agenda 2030. It secures them with an instrument and corresponding methods and approaches to collect and analyze data.

Having said this, while this study addressed top-level policymakers at the MOEHE, it is recommended to run a parallel study involving school leaders themselves. It would be interesting to get answers for the stated research questions, through the lenses of school leaders themselves, and see how they compare with that of senior level policymakers. Perhaps any discrepancy that might be observed can be analyzed for the purpose of supporting school improvement initiatives.

## Footnotes

1 https://www.edu.gov.qa.

2 https://worldpopulationreview.com/countries/qatar-population.

## References

[CR1] Abou-El-Kheir, A. (2017). Qatar’s K-12 education reform—A review of the policy decisions and a look to the future. OSF Preprints. 10.17605/OSF.IO/9U8CA.

[CR2] Acton, K. S. (2021). School leaders as change agents: Do principals have the tools they need? *Management in Education,**35*(1), 43–51.

[CR3] Alshahrani, K., & Ally, M. (Eds.). (2016). *Transforming education in the Gulf region: Emerging learning technologies and innovative pedagogy for the 21st century*. Routledge.

[CR4] Anderson, G. (2021). Turn around Schools: Towards Authentic School Reform: Eroding Authenticity and the Need for Advocacy. *Critical Transformative Educational Leadership and Policy Studies-A Reader: Discussions and Solutions from the Leading Voices in Education*.

[CR5] Bass, B. M., & Riggio, R. E. (2006). *Transformational leadership*. Psychology press.

[CR6] Bellei, C., Contreras, M., Ponce, T., Yañez, I., Díaz, R., & Vielma, C. (2022). The fragility of the school-in-pandemic in Chile. In *Primary and Secondary Education during Covid-19* (pp. 79–103). Springer, Cham.

[CR7] Berkovich, I. (2016). School leaders and transformational leadership theory: time to part ways? *Journal of Educational Administration.,**54*, 609.

[CR8] Berkovich, I., & Benoliel, P. (2021). Framing the role of the school leader in OECD documents: A critical analysis. *Globalisation, Societies and Education,**19*(1), 41–54.

[CR9] Brooks, J. S., & Normore, A. H. (2017). *Foundations of educational leadership: Developing excellent and equitable schools*. Routledge.

[CR10] Bush, T., & Middlewood, D. (2013). *Leading and managing people in education*. Sage.

[CR11] Cairney, P., & Kippin, S. (2022). The future of education equity policy in a COVID-19 world: A qualitative systematic review of lessons from education policymaking. *Open Research Europe,**1*(78), 78. 37645089 10.12688/openreseurope.13834.2 PMC10445953

[CR12] Carrington, S., Spina, N., Kimber, M., Spooner-Lane, R., & Williams, K. E. (2022). Leadership attributes that support school improvement: a realist approach. *School Leadership & Management,**42*, 1–19.

[CR13] Castillo, F. A., & Hallinger, P. (2018). Systematic review of research on educational leadership and management in Latin America, 1991–2017. *Educational Management Administration & Leadership,**46*(2), 207–225.

[CR14] Council of the European Union. (2017). *Council Conclusions on school development and excellent teaching*. 14206/17. data.consilium.europa.eu/doc/document/ST-14206–2017-INIT/en/pdf

[CR15] Day, C., & Sammons, P. (2016). *Successful school leadership*. England: Education Development Trust.

[CR16] Doherty, J. (2021). Teacher leadership as a model of school leadership and professional development. *Profession,**18*, 19.

[CR17] Drisko, J. W., & Maschi, T. (2016). *Content analysis*. Oxford University Press.

[CR18] Ellili-Cherif, M., Romanowski, M. H., & Nasser, R. (2012). All that glitters is not gold: Challenges of teacher and school leader licensure licensing system in Qatar. *International Journal of Educational Development,**32*(3), 471–481.

[CR19] Evans, L. (2022). Is educational leadership (still) worth studying? An epistemic worthiness-informed analysis. *Educational Management Administration & Leadership,**50*(2), 325–348.

[CR20] Fink, E., & Resnick, L. B. (2001). Developing principals as instructional leaders. *Phi Delta Kappan,**82*(8), 598–610.

[CR21] Fowler, F. J., Jr. (2013). *Survey research methods*. Sage publications.

[CR22] Ghamrawi, N., & Tamim, M. R. (2022). A typology for digital leadership in higher education: The case of a large-scale mobile technology initiative (using tablets). *Education and Information Technologies*. 10.1007/s10639-022-11483-w 10.1007/s10639-022-11483-w PMC9707214 36465422

[CR23] Ghamrawi, N. (2011). Trust me: Your school can be better—A message from teachers to principals. *Educational Management Administration & Leadership,**39*(3), 333–348.

[CR24] Ghamrawi, N. (2013). Never underestimate the power of the sandwiched: Middle leaders and school culture. *Basic Research Journal of Educational Research and Review,**2*(2), 29–41.

[CR25] Gillard, D. (2018). Education in England: A history. *Education in England* [Online] May. Retrieved from: www.bit.ly/33t4VQM

[CR26] Gómez-Leal, R., Holzer, A. A., Bradley, C., Fernández-Berrocal, P., & Patti, J. (2022). The relationship between emotional intelligence and leadership in school leaders: A systematic review. *Cambridge Journal of Education,**52*(1), 1–21.

[CR27] Gonzales, M. M., Bickmore, D. L., & Roberts, M. B. (2022). Implementing school improvement plans: Perceptions and implications of aspiring principals for educational leadership programs. *Journal of Research on Leadership Education,**17*(2), 160–180.

[CR28] Gronn, P. (2003). *The new work of educational leaders: Changing leadership practice in an era of school reform*. Sage.

[CR29] Gumus, S., Bellibas, M. S., Esen, M., & Gumus, E. (2018). A systematic review of studies on leadership models in educational research from 1980 to 2014. *Educational Management Administration & Leadership,**46*(1), 25–48.

[CR30] Gunter, H. M. (2016). *An intellectual history of school leadership practice and research*. Bloomsbury Publishing.

[CR31] Guthrie, C., Santos, A. V. P. E., Henderson, K., Norfolk-Beadle, A., Fordham, E., & Baucal, A. (2022). Strengthening evaluation capacity to support the most at-risk schools and build school leadership.

[CR32] Hallinger, P. (2010). Developing instructional leadership. In *Developing successful leadership* (pp. 61–76). Springer, Dordrecht.

[CR33] Hallinger, P. (2003). Leading educational change: Reflections on the practice of instructional and transformational leadership. *Cambridge Journal of Education,**33*(3), 329–352.

[CR34] Hallinger, P. (2015). *Assessing instructional leadership with the principal instructional management rating scale with Chia-Wen Chen and Dongyu Li*. Springer.

[CR35] Harris, A. (2013). *Distributed leadership matters: Perspectives, practicalities, and potential*. Corwin Press.

[CR36] Hatcher, R. (2005). The distribution of leadership and power in schools. *British Journal of Sociology of Education,**26*(2), 253–267.

[CR37] Hayes, S. D., & Irby, B. J. (2020). Challenges in preparing aspiring principals for instructional leadership: Voices from the field. *International Journal of Leadership in Education,**23*(2), 131–151.

[CR38] Heck, R. H., & Hallinger, P. (2009). Assessing the contribution of distributed leadership to school improvement and growth in math achievement. *American Educational Research Journal,**46*(3), 659–689.

[CR39] Heffernan, A., MacDonald, K., & Longmuir, F. (2022). The emotional intensity of educational leadership: A scoping review. *International Journal of Leadership in Education*. 10.1080/13603124.2022.2042856

[CR40] Hendricks, M., & Scheerens, J. (2013). School leadership effects revisited: A review of empirical studies guided by indirect-effect models. *School Leadership and Management,**33*(4), 373–394.

[CR41] Hopkins, D. (2015). *Improving the quality of education for all: A handbook of staff development activities*. Routledge.

[CR42] Jambo, D., & Hongde, L. (2020). The effect of principal’s distributed leadership practice on students’ academic achievement: A systematic review of the literature. *International Journal of Higher Education,**9*(1), 189–198.

[CR43] Jerrim, J., & Sims, S. (2022). School accountability and teacher stress: International evidence from the OECD TALIS study. *Educational Assessment, Evaluation and Accountability,**34*(1), 5–32.

[CR44] Ketikidou, G., & Saiti, A. (2022). The promotion of inclusive education through sustainable and systemic leadership. *International Journal of Leadership in Education*. 10.1080/13603124.2022.2032368

[CR45] Kyngäs, H., & Kaakinen, P. (2020). Deductive content analysis. In H. Kyngäs, K. Mikkonen, & M. Kääriäinen (Eds.), *The application of content analysis in nursing science research. *Cham: Springer.

[CR46] Harris, A., & Jones, M. S. (Eds.). (2015). *Leading futures: Global perspectives on educational leadership. *SAGE Publications India.

[CR47] Leithwood, K. (2021). A review of evidence about equitable school leadership. *Education Sciences,**11*(8), 377.

[CR48] Leithwood, K., Harris, A., & Hopkins, D. (2020). Seven strong claims about successful school leadership revisited. *School Leadership & Management,**40*(1), 5–22.

[CR49] Leithwood, K., & Mascall, B. (2008). Collective leadership effects on student achievement. *Educational Administration Quarterly,**44*(4), 529–561.

[CR50] Leithwood, K., Mascall, B., & Strauss, T. (2009). *Distributed leadership according to the evidence*. Routledge.

[CR51] Liu, Y., Bellibaş, M. Ş, & Gümüş, S. (2021). The effect of instructional leadership and distributed leadership on teacher self-efficacy and job satisfaction: Mediating roles of supportive school culture and teacher collaboration. *Educational Management Administration & Leadership,**49*(3), 430–453.

[CR52] Lochmiller, C. R. (2018). Coaching principals for the complexity of school reform. *Journal of School Leadership,**28*(2), 144–172.

[CR53] Ma, X., & Marion, R. (2021). Exploring how instructional leadership affects teacher efficacy: A multilevel analysis. *Educational Management Administration & Leadership,**49*(1), 188–207.

[CR54] Mac Ruairc, G. (2010). This way please! Improving school leadership: The OECD way. *Journal of Educational Administration and History,**42*(3), 223–246.

[CR55] Mayger, L. K., & Provinzano, K. (2022). Community school leadership: Identifying qualities necessary for developing and supporting equity-centered principals. *Leadership and Policy in Schools,**21*(2), 281–302.

[CR56] McKibben, W. B., Cade, R., Purgason, L. L., & Wahesh, E. (2020). How to conduct a deductive content analysis in counseling research. *Counseling Outcome Research and Evaluation*, 1–13.

[CR57] Mette, I. M., & Scribner, J. P. (2014). Turnaround, transformational, or transactional leadership: An ethical dilemma in school reform. *Journal of Cases in Educational Leadership,**17*(4), 3–18.

[CR58] Meyer, A., Richter, D., & Hartung-Beck, V. (2022). The relationship between principal leadership and teacher collaboration: Investigating the mediating effect of teachers’ collective efficacy. *Educational Management Administration & Leadership,**50*(4), 593–612.

[CR59] Michelsen, G., & Wells, P. J. (2017). *A Decade of progress on education for sustainable development: reflections from the UNESCO Chairs Programme*. UNESCO Publishing.

[CR60] Myran, S., & Sutherland, I. (2019). Defining learning in educational leadership: Reframing the narrative. *Educational Administration Quarterly,**55*(4), 657–696.

[CR61] Nasser, R. (2017). Qatar’s educational reform past and future: Challenges in teacher development. *Open Review of Educational Research,**4*(1), 1–19.

[CR62] Neumerski, C. M. (2013). Rethinking instructional leadership, a review: What do we know about principal, teacher, and coach instructional leadership, and where should we go from here? *Educational Administration Quarterly,**49*(2), 310–347.

[CR63] Nolan, L. (2012). *Liberalizing monarchies: How Gulf monarchies manage education reform*. Doha: Brookings Doha Center.

[CR64] OECD. (2016). Overview: Excellence and Equity in Education. In *PISA 2015 Results (Volume I): Excellence and Equity in Education*. Paris: OECD Publishing. 10.1787/9789264266490-5-en.

[CR65] Özdemir, N., Kılınç, A. Ç., & Turan, S. (2022). Instructional leadership, power distance, teacher enthusiasm, and differentiated instruction in Turkey: Testing a multilevel moderated mediation model. *Asia Pacific Journal of Education*. 10.1080/02188791.2022.2084361

[CR66] Pont, B., Moorman, H., & Nusche, D. (2008). *Improving school leadership* (Vol. 1, pp. 1–199). Paris: OECD.

[CR67] PricewaterhouseCoopers. (2007). Independent Study into School Leadership: Main Report, Department for Education and Skills, London, England.

[CR68] QNB. (2022). *Education Sector in Qatar*. Retrieved from: https://www.qdb.qa/ar/Documents/Education_Sector_in_Qatar_AR.pdf

[CR69] Ranson, S., Arnott, M., McKeown, P., Martin, J., & Smith, P. (2005). The participation of volunteer citizens in school governance. *Educational Review,**57*(3), 357–371.

[CR70] Romanowski, M. H., & Du, X. (2020). Education transferring and decentralized reforms: The case of Qatar. *Prospects*, 1–14.

[CR71] Schechter, C., Shaked, H., Ganon-Shilon, S., & Goldratt, M. (2018). Leadership metaphors: School principals’ sense-making of a national reform. *Leadership and Policy in Schools,**17*(1), 1–26.

[CR72] Schleicher, A. (2015). *Schools for 21st-century learners: Strong leaders, confident teachers, innovative approaches international summit on the teaching profession*. Paris: OECD Publishing.

[CR73] Schröder, J. (2016). Face-to-face surveys. GESIS survey guidelines. Mannheim, Germany: GESIS – Leibniz Institute for the Social Sciences. 10.15465/gesis-sg_en_005

[CR74] Slaughter, M. (2012). Why Women Can’t Have It All. The Atlantic. Retrieved from http://www.theatlantic.com/magazine/archive/2012/07/why-women-still-can-8217-t-have-it-all/9020/#.UAssVeObGhM.email

[CR75] Smylie, M. A., & Murphy, J. (2018). School leader standards from ISLLC to PSEL: Notes on their development and the work ahead. *UCEA Review*, *24*.

[CR76] Sortkaer, B., & Reimer, D. (2018). Classroom disciplinary climate of schools and gender–evidence from the Nordic countries. *School Effectiveness and School Improvement,**29*(4), 511–528.

[CR77] Spillane, J. P. (2005). Distributed leadership. In *The educational forum* (Vol. 69, No. 2, pp. 143–150). Taylor & Francis Group.

[CR78] Stemler, S. E. (2015). Content analysis. *Emerging trends in the social and behavioral sciences: An Interdisciplinary, Searchable, and Linkable Resource*, 1–14.

[CR79] Stevens, J. (2022). Transformational and transactional leadership: An analysis of the leader-follower relationship and their influence in the workplace.

[CR80] Sudibjo, N., & Prameswari, R. K. (2021). The effects of knowledge sharing and person–organization fit on the relationship between transformational leadership on innovative work behavior. *Heliyon,**7*(6), e07334. 34195436 10.1016/j.heliyon.2021.e07334 PMC8239725

[CR81] Sun, J., & Leithwood, K. (2015). Direction-setting school leadership practices: A meta-analytical review of evidence about their influence. *School Effectiveness and School Improvement,**26*(4), 499–523.

[CR82] Tan, C. Y. (2022). Influence of cultural values on Singapore school leadership. *Educational Management Administration & Leadership*. 10.1177/17411432211073414

[CR83] Tan, C. Y., Gao, L., & Shi, M. (2022). Second-order meta-analysis synthesizing the evidence on associations between school leadership and different school outcomes. *Educational Management Administration & Leadership,**50*(3), 469–490.

[CR84] Thien, L. M., & Chan, S. Y. (2022). One-size-fits-all? A cross-validation study of distributed leadership and teacher academic optimism. *Educational Management Administration & Leadership,**50*(1), 43–63.

[CR85] Thomson, S., & Hillman, K. (2019). The Teaching and Learning International Survey 2018. Australian Report Volume 1: Teachers and School Leaders as Lifelong Learners.

[CR86] Townsend, T. (2019). Changing understandings of school leadership. In *Instructional Leadership and Leadership for Learning in Schools* (pp. 1–12). Palgrave Macmillan, Cham.

[CR87] UNESCO (2016). *Leading better learning: School leadership and quality in the Education 2030 agenda*. *Regional reviews of policies and practices*. Retrieved September 22, 2020, from http://www.UNESCO.org/new/fileadmin/MULTIMEDIA/HQ/ED/pdf/abstract-Leadership.pdf

[CR88] UNICEF. (2020). UNESCO Institute for Statistics. Retrieved from: www.data.uis.UNESCO.org/

[CR89] Velarde, J. M., Ghani, M. F., Adams, D., & Cheah, J. H. (2022). Towards a healthy school climate: The mediating effect of transformational leadership on cultural intelligence and organisational health. *Educational Management Administration & Leadership,**50*(1), 163–184.

[CR90] Von Hippel, C., Wiryakusuma, C., Bowden, J., & Shochet, M. (2011). Stereotype threat and female communication styles. *Personality and Social Psychology Bulletin,**37*(10), 1312–1324. 21646549 10.1177/0146167211410439

[CR91] Watson, L. (2009). Issues in reinventing school leadership: Reviewing the OECD report on improving school leadership from an Australian perspective. *Leading and Managing,**15*(1), 1–13.

[CR100] Yazan, B. (2015). Three approaches to case study methods in education: Yin, Merriam, and Stake. *The Qualitative Report*, *20*(2), 134–152. 10.46743/2160-3715/2015.2102

[CR92] Zhang, X., Kuchinke, L., Woud, M. L., Velten, J., & Margraf, J. (2017). Survey method matters: Online/offline questionnaires and face-to-face or telephone interviews differ. *Computers in Human Behavior,**71*, 172–180.

